# Evaluation-Dependent Representation in Risk Defusing

**DOI:** 10.3389/fpsyg.2017.00836

**Published:** 2017-05-23

**Authors:** Oswald Huber

**Affiliations:** Department of Psychology, University of FribourgFribourg, Switzerland

**Keywords:** decision making, risky decision making, representation, evaluation, risk defusing, risk defusing operator (RDO), advantages first principle

This paper treats the effect of the evaluation of outcomes on the representation in a decision process. I assume that how the outcomes are evaluated up to a specific step affects the representation in this step. Thus, representation and evaluation in the process are intermingled.

Research in the tradition of Psychological decision theory investigates risky decisions in experiments generally using gambles as alternatives, or alternatives that are designed like gambles by the experimenter. A gamble is characterized by its outcomes (gains, losses) and their probabilities, all these are known to the decision maker. The fundamental influences determining decision behavior in such experiments are the subjective values (utilities) of the outcomes and frequently their subjective probability. The most prominent decision theories founded in the gambling paradigm are Subjectively Expected Utility theory and its descendants, e.g., Prospect theory Kahneman and Tversky (1979), Baron (2008) gives an overview. Also, decision heuristics are based on one or more of these components (Shah and Oppenheimer, 2008). Most papers in this review paper are based on process tracing methods (e.g., alternatives × dimensions-matrix, verbal protocols).

If in experiments realistic scenarios are used instead of gambles, decision behavior differs in two respects: First, decision makers are often content with knowing whether a certain outcome occurs with certainty or is possible, and are not actively interested in more precise probabilities. Second, decision makers often actively seek a risk defusing operator which reduces the risk.

A *risk-defusing operator* (*RDO*) is an action that is anticipated to remove or reduce the risk. This action is planned by the decision maker to be carried out *in addition* to an existing alternative. Consider, for example, the situation of a person who thinks about the action alternative to travel into a country where a contagious illness endures (Huber, 2012). This person may inquire, for example, whether a vaccination exists. Getting vaccinated is an RDO preventing the negative outcome (infection). If a satisfactory RDO is found with an otherwise attractive alternative, this alternative is usually chosen (e.g., Bär and Huber, 2008). An overview about the decision process and experimental results concerning RDOs is presented in Huber (2007, 2012).

There are various types of RDOs (e.g., Huber, 2007): one type *prevents* a negative outcome (e.g., vaccination, drinking bottled water only), for example, or another *compensates* for a negative outcome (e.g., insurance). Search for an RDO often involves a price (money, time, effort, etc.), and at the start it is unclear whether the search turns out to be effective. Understandably, search is more likely if the expectation of success is higher (Huber and Huber, 2008). Search is also more likely under time pressure (Huber and Kunz, 2007) and under justification pressure (Huber et al., 2009). Moreover, the type of risk influences the search (Wilke et al., 2008). An RDO is not automatically satisfactory: The higher the cost, the less likely is the RDO accepted (Williamson et al., 2000; Huber and Huber, 2003). In a multistage investment task too, people are willing to purchase an RDO, if they are given the opportunity (Huber, 1996).

The conception of RDOs is overlooked in classical decision research. This disregard seems to result from the belief that *all* risky decisions involve gambles, where RDOs are not relevant. Gambles can be considered to form only a subclass of risky decision tasks.

## Dynamic mental representation of alternatives

Classical descriptive decision theory is based on Subjective Expected Utility Theory and its modifications. In these theories, the representation of alternatives is rarely addressed explicitly. Prospect theory (Kahneman and Tversky, 1979) is an exception. The authors suppose that representation is a distinct first phase of the decision process, and then—in a second phase—the alternatives are evaluated.

In the risk-defusing context, a different approach is taken. Decision makers in non-routine situations are assumed to construct a mental representation by sequentially incorporating new information they consider as relevant (outcomes, probabilities, RDOs, …) into a causal mental model of the alternatives (Huber, 2011). The representation is *dynamic*, not only because it is constructed in time, but also because in may be changed (by introducing an RDO) in the course of elaboration. These mental models usually do include a different amount of elements for different alternatives: more information is represented for some alternatives, less for others. I assume that normally items like outcomes are evaluated immediately when they are introduced into the representation as more or less desirable or undesirable. How much is represented for each alternative, depends on this evaluation, as described in the next section. Thus, elaboration and evaluative processes are closely intermingled (in contrast to, e.g., Prospect theory).

## Advantages first principle

We have shown in three experiments that the great majority of decision makers follow the Advantages first Principle (Huber et al., 2011). This principle describes, how information search is guided by the evaluation up to now:

1. Decision makers consider mainly outcomes evaluated as positive, and based on this evaluation, promising alternatives are distinguished from not promising ones.
2. They then inspect the promising alternatives further and deliberately search—among others—for undesirable outcomes^1^. Of course, when they detect a negative outcome they may search for an RDO.

Thus, the Advantages first Principle is not a choice heuristic (selecting *the* subjectively best alternative) but a heuristic to select promising problem solving paths. The situation is similar to chess, where expert players do not examine every possible move. Instead, they center on few moves that seem worth pursuing founded on a preliminary evaluation (Holding, 1992). The early search for positive or negative outcomes when decision makers have no previous information has not been studied systematically in decision research, to the best of my knowledge.

The decision maker concentrates on positive outcomes and attractive alternatives because elaboration is expensive in time, effort, etc., and he or she wants to reduce these cost. (1) If an alternative has an attractive positive outcome, time and effort can be invested into this alternative to examine it in more detail. A negative outcome may be defused with an acceptable RDO. (2) If, however, the positive outcome of an alternative is only mediocre, then it remains only mediocre, even though no negative outcome should turn up. (3) Concentrating initially on the negative outcomes would not be an economical heuristic to decide which alternatives to inspect more. An exception is an alternative with a very negative outcome which cannot be defused. In this case, it can be ignored and no information has to be searched for. In all other situations, it would always be essential to also check the positive outcomes. Otherwise, one might invest much time and effort in an alternative that later turns out to be inferior.

To recapitulate, the Advantages first Principle defines a rational heuristic for selecting alternatives deserving a more careful inspection. The decision maker can generally at all times return to an alternative that he or she thinks should have been examined deeper.

As mentioned above, the majority of decision makers uses the Advances first Principle (more than 80%), but a minority investigated negative outcomes first. Huber et al. (2011) presume that the principle is applied, when (a) decision makers are free to acquire the information in the order they prefer, (b) they do not already have exhaustive knowledge about the alternatives (they are no experts), (c) they do not assume that the set of available alternatives is a (positive) pre-selection with good positive outcomes for all alternatives, and (d) there is no acceptance criterion that all alternatives have to fulfill (like the maximum rent). Furthermore, in time pressure conditions, most people start with a negative outcome, but without time pressure, they start with a positive one (Huber and Kunz, 2007). Time pressure here means that there is a kind of (external or internal) deadline *and* the decision maker realizes that the available time may be too short to make a decision (e.g., Benson and Beach, 1996).

The Advantages first Principle is, as stated above, a heuristic for selecting a promising problem solving path. So possibly an alternative picked out first is not chosen, e.g., because a negative outcome cannot be defused. This is not a falsifying instance for Advantages first, because it does not predict choices. There should, though, be a correlation between selecting a promising alternative and the chosen one, albeit we do not know at present how big this correlation is. Therefore, it will be essential to investigate the research that found negative outcomes to have a more pronounced effect, as, for example, the framing effect for gains (Tversky and Kahneman, 1981), or Priority heuristic (Brandstätter et al., 2006).

## Representation—positive and negative outcomes

Up to now, we have only considered RDOs defusing a negative outcome (*negative* RDOs). An RDO may, however, improve the chance to receive a positive outcome (*positive* RDOs). An example is doping in sports, which increases the chances of winning. The question I want to address here is whether positive RDOs are searched for equally often as negative ones.

We investigated this question by embedding the decisions in a framing context, see Huber et al. (2014) for details. Otherwise we are confronted with the problem that frames causing search for either positive or for negative RDOs often involve outcomes with distinct attractiveness, and—as the previous section clarified—attractiveness is one of the central factors influencing RDO search. The result was clear: Much more people (83%) searched for a negative RDO, whereas only 39% searched for a positive one. We attribute this result to a general readiness for negative stimuli and for starting adequate reactions (Taylor, 1991; Öhman et al., 2000; Woody and Szechtman, 2013). Such readiness is favorable because if somebody is provoked with a possible danger (e.g., a person aiming a pistol on me, or a snake), it is frequently vital to react fast. Positive stimuli are not connected with an analogous general readiness, at least nothing comparable is reported in the literature. Searching for an RDO preventing or compensating the negative stimulus clearly is such an adequate reaction when confronted with a possible negative outcome.

Thus, negative RDOs are activated or investigated more often when otherwise a negative alteration is possible than positive ones when a positive alteration is possible. So, in this case too, evaluation of the alternatives is a factor determining the construction of a representation.

## Conclusion

The previous sections have demonstrated that how the outcomes are evaluated is a central aspect in the process of constructing a representation. I want to empathize I expect the Advantages First Principle to hold in what we could call non-routine situations, as described in the relevant section. The Advantages first Principle is explicitly not a choice heuristic, and does not predict that choice is based solely on positive outcomes. It is, to repeat, a heuristic selecting a path that is worthwhile to be followed.

I could not go into details of theories that would be useful to be investigated, for example, Naturalistic decision making and Query theory. Naturalistic decision making (Klein, 1999) deals with realistic decision situations. It is, however, concerned mainly with non-experimental research in decisions of experts, and experts are explicitly not the topic of my paper. Query theory (e.g., Johnson et al., 2007) proposes people to construct their values. The used method is interesting and seems to be a variant of thinking aloud.

By concentrating on evaluation I did of course not want to exclude other influences on risk defusing. Such effects are, for example: justification pressure, the expectation of search success, the type of risk involved, or the expectation to get useful probability information. However, an inclusion of these topics is beyond the scope of this paper.

## Author contributions

The author confirms being the sole contributor of this work and approved it for publication.

### Conflict of interest statement

The author declares that the research was conducted in the absence of any commercial or financial relationships that could be construed as a potential conflict of interest.

## Footnotes

^1^This result speaks against Montgomery and Willén (1999) *dominance structuring* model. For a more detailed discussion, see Huber (2011).
